# Performance Optimization of Microvalves Based on a Microhole Array for Microfluidic Chips

**DOI:** 10.1155/2020/8842890

**Published:** 2020-09-03

**Authors:** Cuimin Sun, Hui You, Yang Xie, Ronald X. Xu

**Affiliations:** ^1^Department of Precision Machinery and Precision Instrumentation, University of Science and Technology of China, Hefei, Anhui 230001, China; ^2^XingJian College of Science and Liberal Arts of Guangxi University, Nanning, Guangxi 530004, China; ^3^School of Mechanical Engineering, Guangxi University, Nanning, Guangxi 530004, China

## Abstract

A microfluidic chip with a microvalve based on a microhole array is proposed in this paper for the POCT of tumor marker proteins. In order to control the biochemical reaction time accurately and obtain a higher testing sensitivity, the parameters of the microhole array are optimized basing on the investigation of the effects of the variation of those parameters on the fluid rate and the residual liquid value in the microvalve region. By conducting liquid flow experiments using microvalves based on microhole arrays with varying microstructural parameters, the residual rate of reaction products is demonstrated to be proportional to the depth and diameter of the microholes and inversely proportional to the distance between the microhole centers. A comprehensive analysis indicates that a microhole depth of 95 *μ*m, a microhole diameter of 230 *μ*m, and a distance between microhole centers of 250 *μ*m not only ensure a sufficiently long delay time, but also reduce the residual rate of reaction products, thereby providing an optimum microvalve performance that maximizes the detection efficiency and accuracy of microfluidic chips.

## 1. Introduction

Most POCT [1] technologies available worldwide for real-time in vitro diagnostic testing have been designed and developed based on microfluidic chips, and these have been generally applied for the detection of substances with a relatively high rate in the body fluids of patients, such as proteins [2] and blood sugar [3]. At present, low accuracy of the flow control and relatively large residual value are the main shortcomings of the microfluidic systems in such POCT chips, which results in inadequate biochemical reaction and large interassay imprecision. These shortcomings mainly come from the microvalves integrated in the chips. Therefore, the further development of the POCT chips is highly dependent on the performance of the microvalves used in the chips. Most microfluidic systems for POCT applications use passive microvalves, especially the capillary microvalves including paper-based capillary microvalves [4], polymer capillary microvalves [5], and microstructured capillary microvalves [6]. Of which, the microstructured capillary microvalves have been addressed by researchers because of their advantages of good biological compatibility, economy, control performance, and ease of manufacturing [7]. The mechanism of this kind of microvalves is based on the phenomenon that the surface parameters of the microstructures, such as contact angle and roughness, affect the capillary force and thus affect the flow state. The surface effect on the flow has been investigated in the channel in different scales. Xu et al. [8] investigated the effect of the wall roughness on the fluid transport resistance of the water inside nanopores and found that the transport resistance was larger when the relative roughness was higher. Pan et al. [9] developed a novel surface composed of a shape memory micropillar array which can tune the wettability of the surface. The as-obtained surface does not only exhibit reversible wettability between superhydrophobic and slightly hydrophobic, but also exhibit repairable superhydrophobicity. Kim and Varenberg [10] reported on the behavior of original and split, wall-shaped adhesive microstructures on different surfaces, and their results clearly demonstrated that the adhesion- and friction-driven attachment of the wall-shaped microstructure degrades when the surface roughness increases. Sarafraz and Hormozi [11] experimentally investigated the influence of some parameters such as roughness of the surface, boiling contact angle, and deposition on the pool boiling heat transfer coefficient of nanofluids. When the concentrate of the nanofluid increased, surface roughness of the heating section and the boiling contact angle of bubbles with the surface significantly decreased. Zhixiong et al. [12] found that the boiling thermal performance of the copper oxide nanosuspension was quantified on the modified surfaces with different geometrical specifications, and the fins design with more towering height and smaller width achieved the best thermal performance.

Many microstructured microvalves based on the capillary force and surface effect have been developed. Du et al. [13] tailored the flow rate through capillary microvalves by adjusting the hydrophilicity of the microchannel via the insertion of deep holes on their interior surfaces, which altered the contact angles between the liquid and the microchannel surfaces to achieve a delay function. Suh et al. [14] proposed a microstructured microvalve based on capillary force, which affects the flow state of the liquid in different sections of the microchannel according to their different cross-sectional areas. Chang et al. [15] presented a novel design of a capillary stop valve with a chamfered side that can be used as a flow regulator to hold an injected microfluid in the valve position in a capillary force-driven microfluidic device.

Up to now, most research studies on the capillary microvalves focused on the flow velocity and the fabrication, and no studies have investigated the residual value in the microvalve structures, which affects the detection sensitivity on the occasion that the microvalves are used in POCT chips. We previously developed a capillary microvalve with the microhole array on one of the interior surfaces of the microchannel and investigated the effects of the parameters of the microhole array on the fluid head velocity [16]. However, the effects of these parameters on the residual value in the microvalve region were not investigated. Basing on the work, a microfluidic chip with a microvalve based on a microhole array is proposed in this paper for the POCT application of the tumor marker protein test. The issue of the residual value in the microvalve region is addressed. In order to control the biochemical reaction time accurately and obtain a higher testing sensitivity, the parameters of the microhole array are optimized basing on the investigation of the effects of the variation of those parameters on the fluid rate and the residual liquid value in the microvalve region.

## 2. Experimental

### 2.1. Microfluidic Chip and Its Procession of Work

**Figure 1:**
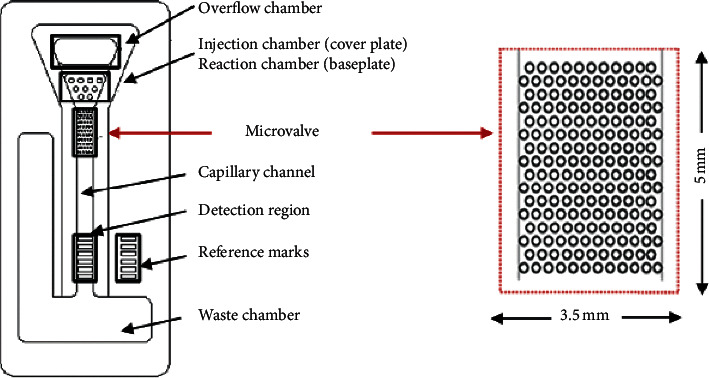
Diagram of a microfluidic chip designed for the detection of tumor markers.

The design of a passive microfluidic chip for the detection of the proteins serving as tumor markers is illustrated in Figure 1, and the proposed passive microvalve based on the microhole array is shown at the right of the figure. When a blood sample is dropped in the injection chamber, the serum penetrates through the filter paper and flows into the reaction region below, where the tumor marker proteins in the serum are labeled with the luminescent material by the immunological reaction. The microvalve region is the short section of the microchannel with a microhole array on the bottom surface, which can maintain the serum within the reaction region for a sufficient period to ensure the completion of the reaction. Then, the serum flows quickly through the microvalve and along the capillary channel to the detection region and then to the waste chamber. When the serum flows past the detection region, the tumor marker proteins contented are specifically captured by the special antibody anchored on the surface of the channel bottom, and therefore the color bars appear due to the luminescent material. The quantity of the tumor marker proteins can be evaluated according to the intensity of the color.

### 2.2. Working Principles of the Microvalve

The microstructured capillary microvalve proposed in this paper has two main functions: one is to maintain the sample fluid within the reaction region for a sufficient period to ensure the completion of the biochemical reactions, while the other function is to enable the sample fluid to quickly flow through with very little liquid remaining after the reaction completion to make the total test time shorter and test sensitivity higher. These two functions are fulfilled by reducing the fluid head velocity as the sample fluid flows into the microvalve region and then allowing the fluid to flow quickly once the microvalve region becomes saturated with the fluid. In this sense, the function of the microvalve is similar to that of a passive slow-opening valve.

**Figure 2:**
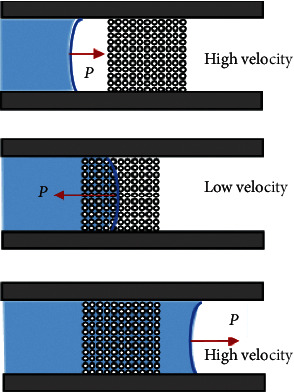
Schematic illustrating the fluid sample flow process through the microvalve based on the microhole array.

The process of the liquid sample flow through the microvalve can be divided into the three stages as shown in Figure 2, where the vector *P* represents the capillary force. The liquid head velocity of the fluid sample varies according to the changes in the hydrophobicity of the microchannel induced by the microhole array.

The microvalve based on the microhole array was designed according to the Wenzel model [17], which is commonly used for predicting the equilibrium contact angle (CA) of drops which penetrate the asperities of a rough surface and do not account for the liquid volume stored in the asperities. As such, the hydrophobicity of the solid surface can be determined according to the contact angle [18]:

(1) $$\begin{array}{c} \cos\theta_{b}=r\cos\theta_{e}, \end{array}$$

where *θ*_*b*_ is the predicted Wenzel contact angle, *r* is the roughness factor of the solid surface, which is given as the rate of the area of the solid-liquid interface and the projected area: *A*_SL_/*A*_projected_, and *θ*_*e*_ is the contact angle of a liquid droplet on a smooth surface made up of the same material (i.e., the contact angle associated with Young's equation). Applying this relationship to the microhole array structure yields the following formula:

(2) $$\begin{array}{c} \cos\theta_{b}=\left(1+\frac{2\pi dh}{a^{2}}\right)\cos\theta_{e}, \end{array}$$

where *h* is the microhole depth, *d* is the microhole diameter, and *a* is the distance between microhole centers. In addition, our previous study established the following equation governing the effect of the microstructural parameters on the flow period [16]:

(3) $$\begin{array}{c} t=k\left(1+\frac{\pi dh}{2a^{2}}\right), \end{array}$$

where *k* is related to various factors such as the liquid viscosity, material properties, and temperature.

The adsorption of fluids on solid surfaces can be divided into physical adsorption and chemical adsorption according to the nature of the binding force between the adsorbed liquid molecules and the atoms on the solid surface. Physical adsorption is produced by Van der Waals forces and is closely related to the surface area and hole size distribution of solids. Chemical adsorption can be produced only under particular conditions due to its chemical bonding nature and includes some level of selectivity. Here, we consider the residual liquid value on the microvalve region only from the perspective of physical adsorption. The well-known Gibbs adsorption isotherm formula can be applied to determine the quantity of a physically absorbed liquid species on a solid as follows [19]:

(4) $$\begin{array}{c} Q=-\frac{c}{RT}\frac{d\gamma }{dc}, \end{array}$$

where *c* is the activity of the component, *γ* is the surface tension, *R* is the ideal gas constant, and *T* is the temperature. As such, the quantity of an absorbed liquid on a solid surface depends expressly on the surface tension. The surface tension is difficult to be measured directly, but it can be obtained from the contact angle *θ*_*b*_ given by formula (2). Therefore, the residual value, due to the adsorption on the surface of the microvalve region, is related to the microhole array parameters *a*, *d*, and *h* according to formulas (2) and (4).

### 2.3. Fabrication of the Microfluidic Chip

**Figure 3:**
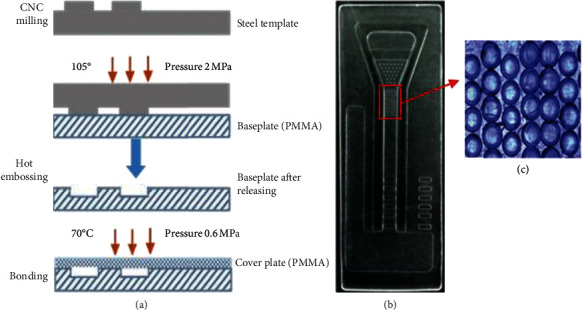
(a) Microfluidic chip fabrication process; (b) an image of a completed chip; (c) the microhole array under magnification.

The manufacturing process of the microfluidic chip is shown in Figure 3. The microfluidic chip consists of a cover plate and a baseplate made of polymethyl methacrylate (PMMA) sheets. The baseplate, including the microchannel and the microvalve, is fabricated with a PMMA sheet by the hot embossing process using a steel template, under the condition of 105°C temperature, 2 MPa pressure, and 90 minutes molding time and cooling slowly after the molding. The steel template with the microchannel and the microvalve pattern was fabricated in advance by CNC milling. The cover plate of the injection chamber is fabricated by CNC milling as well. The base- and cover plates of the microfluidic chip are rinsed with deionized water for 10 minutes in an ultrasonic cleaner at room temperature and then subjected to plasma cleaning to improve their surface activity. Finally, the two plates are fused together under a pressure of 0.6 MPa and a temperature of 70°C for 20 minutes.

### 2.4. Microvalve Performance Testing

A series of the microfluidic chips with the same channel size (3.5 mm in width, 105 *μ*m in depth, and 35 mm in length) and different sets of the parameters of the microhole array were fabricated and tested with DI water. In the chips, the areas of the microhole arrays were the same (3.5 mm wide and 5 mm long), but the diameter of the holes *d* varied from 170 to 240 *μ*m by 10 *μ*m, while the depth of the holes *h* varied from 60 to 100 *μ*m by 5 *μ*m, and the distance of microholes *a* varied from 225 to 400 *μ*m by 25 *μ*m. Such ranges of the microholes size were selected because the microvalve in this paper was designed for a commercial product of the POCT chip, which must be easily fabricated on a large scale and the manufacture cost must be low enough, and that was why hot embossing process was used. Limited by the equipment of the process and the manufacture cost, the diameter of the microhole and the distance between microholes cannot be made very smaller, and the microhole cannot be made very deep. On the contrary, the microholes cannot be designed large, otherwise the residual value will be too much. If they are too shallow or the distance between microholes is too large, the surface will be approximately planar, and the regulation of the hydrophilic will be too weak.

The flowing state and residual value tests on five chips of each set of parameters of the microhole array were carried out under a microscope. To avoid the residual value calculation error due to the absorption of the filter paper, there was no filter paper in any chip in the test. 40 *μ*l of DI water doped with the blue ink as the test sample was directly pipetted into the reaction chamber of each chip, and then the fluid head velocities in the microhole array area and the microchannel downstream were measured. The fluid head velocity through the microvalve was calculated according to the period between the moments of the fluid head moving in and out of the microhole area. The fluid head velocity through the microchannel downstream with a length of 30 mm was measured with the same method.

The sample residue of each microvalve is difficult to be measured directly. Here, it was estimated from the photograph of the whole microfluidic system including the waste chamber, which was taken after the end of flowing. The surfaces of chip where flowed past, including the microvalve region, were stained in blue due to their absorption of the ink particles. The quantity of the ink particles absorbed in each region was in proportion to the gray value in the area in the photograph. Therefore, the sample residue rate in the microvalve could be calculated by the rate of the gray value in the region against the total gray value in the whole microfluidic system area.

## 3. Results and Discussion

### 3.1. Fluid Head Velocities of Microfluidic Chips

**Figure 4:**
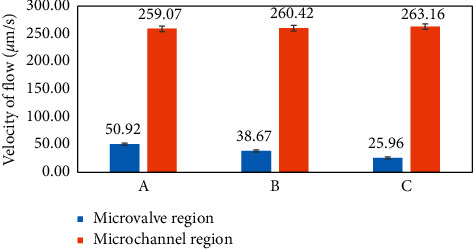
Effects of different microvalve structural parameters on fluid head velocities in the microvalve and microchannel areas.

The fluid head velocities of three types of chips with the same microchannel size (3.5 mm in width, 105 *μ*m in depth, and 35 mm in length) and different sets of microhole array parameters as shown in Table 1 were tested, and the results are compared in Figure 4.

For different types of chips, the head flow velocities in the microvalve regions were significantly different, while the flow velocities in the capillary channels downstream are nearly equivalent. The tests indicated that the fluid head moved very slowly through the microvalve region and then moved much faster in the capillary channel downstream. The flow velocity difference between the two regions was at least 5 times. The lowest fluid head velocity in the microvalve region, which is attained by Chip C, is less than 1/10 of that in the capillary channel downstream. The phenomenon confirms that the microhole array has a significant delay effect on the fluid head when it was moving through the region, but this delay effect immediately disappeared once the liquid head passed the region.

From another perspective, these results show that although the microhole array region was only 5 mm long, most sample liquid was blocked in the reaction chamber for quite a long time (98.2, 129.3, and 192.6 seconds in Chips A, B, and C, respectively) by the fluid head which was moving through the region very slowly. Once the liquid head passed the microhole array region, the sample liquid flows through the channel downstream, which was 30 mm long, within 116 seconds. Accordingly, the microhole array in each chip actually worked as a passive slow-opening valve.

For the chips with different sets of microhole array parameters, the head flow velocities in the microvalve regions were significantly different, while the flow velocities in the capillary channels downstream are nearly equivalent. It indicates that the structural parameters of the microhole array effect its function as a passive slow-opening valve.

### 3.2. Contact Angle and Liquid Residue Rate

**Figure 5:**
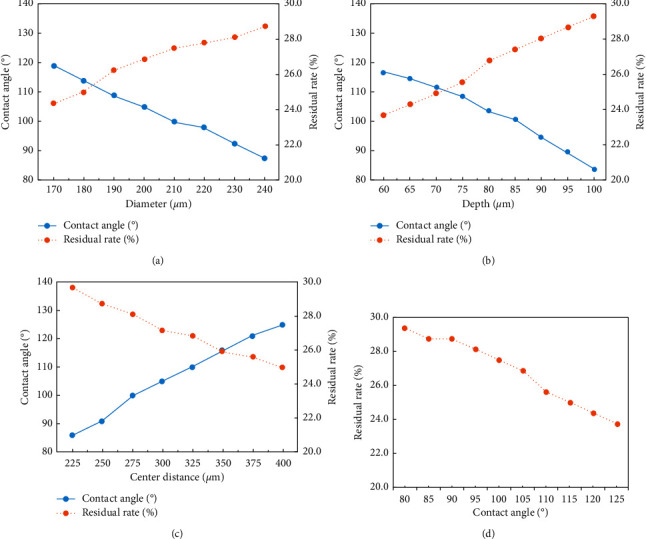
Effects of microstructural parameters on the contact angle and the residual rate of the absorbent in microfluidic chips. (a) Effect of the diameter of the microhole on the results and *h* = 95 *μ*m and *a* = 250 *μ*m. (b) Effect of the depth of the microhole on the results and *d* = 230 *μ*m and *a* = 250 *μ*m. (c) Effect of the center distance of the microholes on the results and *d* = 230 *μ*m and *h* = 95 *μ*m. (d) Relationship between the residual rate and the contact angle results.

The effects of the microhole parameters on the contact angle and the liquid residue rate are shown in Figure 5. As shown in Figures 5(a) and 5(b), when the diameter (*d*) or the depth (*h*) of the microhole increases, the contact angle of the surface decreases, while the liquid residual rate on it is increased. A longer distance between the microhole centers results in a bigger contact angle and a smaller residual rate as shown in Figure 5(c). Basing on Figures 5(a)–5(c), a negative correlation relationship between the contact angle and the liquid residual rate was obtained, which is shown in Figure 5(d). The increase in the contact angle means there is an increase in the surface hydrophobicity, which results in a smaller contact area between the liquid and the surface of the microchannel; therefore, less liquid is absorbed by the surface. The experimental results coincide with our theoretical analyses and can be employed for optimizing the microstructural parameters of the microvalve.

### 3.3. Optimization of Microstructural Parameters for the Best Microvalve Performance

The microvalve performance lies in the three respects: the delay time, the flow speed after the delaying time, and the liquid residue rate in the microhole array region, which are related to the chip functions in the adequacy of the biochemical reaction, total test time, and sensitivity, respectively. We tried to optimize the microhole array design to get the best valve performance in the three respects. As shown in Figure 4, different designs of microhole array parameters almost have no influence on the flow speed after the delaying time. Therefore, the optimization is only aimed to get longer delay time and lower liquid residue rate.

**Figure 6:**
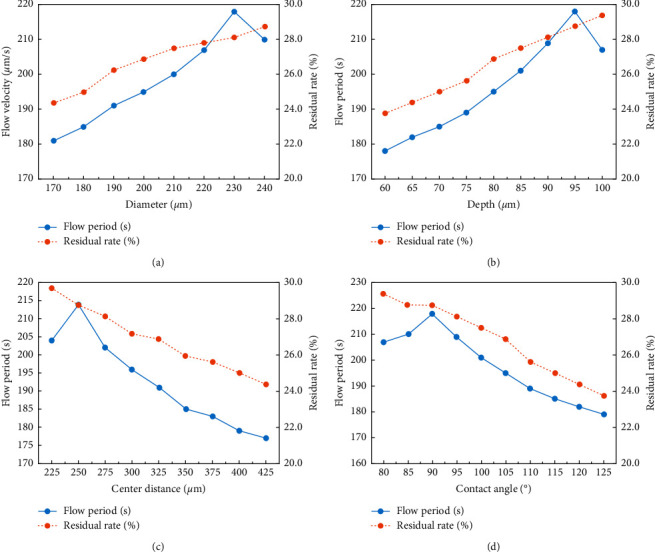
Effects of microstructural parameters on the flow period and the residual rate of the absorbent in microfluidic chips. (a) Effect of the diameter of the microhole on the results and *h* = 95 *μ*m and *a* = 250 *μ*m; (b) effect of the depth of the microhole on the results and *d* = 230 *μ*m and *a* = 250 *μ*m; (c) effect of the center distance of the microholes on the results and *d* = 230 *μ*m and *h* = 95 *μ*m; (d) relationship between the delay time and the contact angle results.

Figures 6(a)–6(c)show the effects of microhole array parameters on the delay time and the liquid residue rate. Within the range of microhole array parameters, we experimented the delay time and the residual rate varied from 179 to 218 seconds and 29.4% to 23.8%, respectively. The variation of the microhole array parameters leads to a similar change trend in the delay time and the residual rate, except that the delay time reaches the peak when the diameter and depth of the hole and the center distance between holes are 230, 95, and 250 *μ*m, respectively, while the residual rate increases monotonously with the increase in those parameters. Therefore, the maximum delay time and the minimum residual rate cannot be achieved simultaneously. We need to balance the two respects to select the most suitable parameters of microhole array parameters. Besides changing the microhole array parameters, the residual rate can also be reduced by increasing the injected sample value. According to the microfluidic chip design, the control of delay time mainly depends on the selection of microhole array parameters. Therefore, the optimization of the microhole array parameters should firstly meet the requirement of the time delay. Since a period of at least 3.5 minutes is required to complete the biomedical reactions in the reaction chamber, the microhole array with 230 *μ*m in diameter, 95 *μ*m in depth, and 250 *μ*m distance between hole centers is selected finally, which leads to the longest delay time of 215 seconds and a relatively not too high residual rate of 28%.

In our previous research, it has been found that the contact angle of the surface with the microhole structure was inversely proportional to the depth and diameter of the microhole and proportional to the distance between the microhole center [16]. In this study, the relationship between the contact angle and the delay time was built as shown in Figure 6(d). The delay time increases with the contact angle when it is less than 90° and decreases as the contact angle increases and when it is greater than 90°. The delay time reached the maximum when the contact angle was around 90°, which coincided with the previous study. Especially, the contact angle is exactly 90° for the surface with the best set of microhole array parameters we selected, which is 230 *μ*m in hole diameter, 95 *μ*m in depth, and 250 *μ*m center distance between holes.

## 4. Conclusions

By conducting liquid flow experiments using microvalves based on microhole arrays with varying microstructural parameters, the residual rate of reaction products is demonstrated to be proportional to the depth and diameter of the microholes and inversely proportional to the distance between the microhole centers. A comprehensive analysis indicates that a microhole depth of 95 *μ*m, a microhole diameter of 230 *μ*m, and a distance between microhole centers of 250 *μ*m not only ensure a sufficiently long delay time, but also reduce the residual rate of reaction products, thereby providing an optimum microvalve performance that maximizes the detection efficiency and accuracy of microfluidic chips. Compared with the traditional microvalve, the microvalve based on the microarray designed in this paper has the following innovations. The accurate control of fluid velocity and the residual rate of reaction products are realized by adjusting the microstructure parameters to meet the requirements of different biochemical reactions on the microfluidic chip. The microvalve with long delay time and small residual rate designed in this paper provides a reference for guiding future research in the optimization of other types of microvalve designs based on the microstructure and also lays a foundation for the design of microfluidic chips used in POCT applications based on these types of microvalves.

The present work employed deionized water as the liquid sample. However, blood plasma typically serves as the liquid sample in practical microfluidic chip applications. Protein adsorption is a complex process that is affected by various factors [20], which mainly include the physical and chemical properties of the proteins and other materials and the influence of the surrounding environment. Although considerable progress has been made in this field, the adsorption mechanisms of proteins require further exploration before a more sophisticated study of the residual rate of reaction products on the inner surface of microvalves can be conducted.

## Figures and Tables

**Table 1:** Three types of microfluidic chips with different sets of microhole array parameters.

Type of chip	*h:* depth of microhole (*μ*m)	*d*: diameter of microhole (*μ*m)	*a*: distance between microhole centers (*μ*m)	*l*: length of microhole array (mm)
Chip A	55	300	400	5
Chip B	75	260	350	5
Chip C	95	230	300	5

## Data Availability

The data used to support the findings of this study are included within the article.
